# Dental Association of Ontario

**Published:** 1867-08

**Authors:** J. S. Scott

**Affiliations:** Cobourg, Canada


### DENTAL ASSOCIATION OF ONTARIO.

BY J. S. SCOTT, M. D., COBOURG, CANADA.

THE members of the Dental Association of Ontario met in the County's Council Chamber at 7 P. M. Present: B. W. Day, M. D., of Kingston, President, in the chair; J. S. Scott, M. D., of Cobourg, and John O'Donnell, of Peterboro, Secretaries; C. S. Chittenden, Hamilton; H. T. Wood, Picton; A. D. Lollonde, Brockville; M. E. Snider, Toronto; F. G. Callendar, Cobourg.

The following Dentists of established office practice of five years and over have filed their certificates as to practice and moral character, were reported by the committee on credentials as eligible for election as active members. They being present, were severally elected, and signed the constitution: Chas. Kahn, Stratford; W. C. Adams, Toronto; L. Lemon, St. Catharine's; Robert Reid, Galt; W. H. Card, Whitby; D. Pentland, Peterboro; J. A. Brown, Port Hope; T. J. Jones, Bowmanville; S. B. Chandler, Newcastle; R. Trotter, Brampton; S. F. Kenedy, Perth; W. H. Porter, Holland Landing; D. A. White, Ridgetown.

The following Dentists having established offices, but being of less than five years' standing, were elected as incipient members. Being present, they severally signed the constitution: J. M. Brimacombe, Bowmanville; J. C. Grass, Windsor; Thos. Rowe, Cobourg; J. R. Irish, Whitby; T. Neelands, Port Hope.

A letter was read from the Rev. S. S. Nelles, D.D., President of Victoria College, inviting the members of the Association to visit the University Buildings to-morrow, at 3 P. M. Accepted.

Adjourned till to-morrow morning at 9 o'clock.

SECOND DAY'S PROCEEDINGS.

The Association met at 9 A. M.

There were thirty-one members in attendance. B. W. Day, M. D., in the chair. J. S. Scott, M. D., and J. O'Donnell, Secretaries.

Letters were read from the following Dentists not in attendance, applying for membership: J. B. Meacham, Brantford; J. Bower, Ingersoll; R. I. Brown, Galt; J. L. Waters, Barrie; G. W. Hawk, Lindsay.

Mr. R. Trotter, Brampton, called the attention of the Association to the requirement of the constitution, exacting that Dentists must have had five years established office practice in order to be eligible for election as active members. He thought the provision good.

Mr. H. T. Wood, Picton, said that the Association, when in session in Toronto, in January last, fully considered the point that an established office practice, which was successful for five years in one place was a fair test of abilities and qualifications of practitioner; that he should regret to see the standard of qualifications lowered, but hoped soon to see it very much raised.

Mr. C. S. Chittenden, Hamilton, would increase rather than lower requirements for membership. He was pleased at meeting so many of the established Dentists of the Province; and hoped we should soon have a Bill passed which would settle the question as to what qualifications should be required of persons practising as Dentists.

J. S. Scott, M. D., said if he could have been certain of so respectable an attendance, he would have taken the liberty of inviting prominent gentlemen of the town to attend as visitors. He would now move, seconded by Mr. C. S. Chittenden, that Corresponding Secretary be requested to invite physicians, clergymen and Cobourg editors, to attend meetings of this session of the Association. Carried.

Thos. Rowe, M. D., stated that he had been requested by the editor of the *Dental Cosmos* to apply for a copy of the proceedings for publication. Granted.

Mr. J. O'Donnell, Secretary of Committee appointed at the Toronto session to draft an Act of incorporation, read a draft of a Bill to incorporate persons practising Dentistry in the Province of Ontario. Ordered to be read a second time this afternoon at two o'clock. At 2 P. M., Bill read a second and third time in Committee of the Whole, and approved.

Mr. J. B. Meacham, Brantford, and Mr. J. Bowers, Ingersoll, were ballotted for and elected as active members. Mr. F. G. Callender, on part of Committee, reported a draft of by-laws, which were adopted.

Mr. C. S. Chittenden read a paper on Dentition, which was well received and ordered to be printed.

G. V. N. Relyea, Belleville, signed the constitution and took his seat, he having been elected at the Toronto session.

Mr. L. Vancamp, Berlin, was elected and took his seat.

Mr. R. Trotter moved, seconded by Mr. C. S. Chittenden, that in the opinion of the Association it is not desirable that the title of Dr. should be applied to Dentists who are not legally entitled to it. Carried.

Dr. G. V. N. Relyea presented to the Association, for inspection, models of irregular teeth which had been straightened by mechanical appliances.

Dr. G. V. N. Relyea, of Belleville, read a paper upon Nitrous Oxide Gas. He stated that he had administered chloroform probably 1,000 times, that he had invariably required that a physician should be in attendance, as it not only served to remove the timidity of the patient, but in case of disagreeable result the best assistance would be at hand. He had had only one case of apparent danger from chloroform; but there was always a risk with regard to its results; and in case anything disagreeable occurred, the presence of a physician would divide the responsibility. He considered nitrous oxide gas preferable; he had administered eighty times during the last nine months with the most satisfactory results. He always required the presence of an assistant; but did not think it necessary to insist upon the presence of a physician; he did not consider it dangerous in the sense in which chloroform was dangerous. In one fatal case referred to—laughing gas—a *post mortem* examination revealed the fact that organic disease existed to an extent, that the removal of a tooth would have proved fatal.

J. S. Scott, M. D., of Cobourg, replied to Dr. Relyea's question, as to his experience with nitrous oxide gas. He had been using it with most satisfactory results for little over a year. He met with difficulty at first in the way of procuring proper apparatus for manufacturing and preserving the gas. It should be kept on hand in a gasometer ready for use. He had administered it nearly a hundred times. He kept a register with the names arranged alphabetically. The best way to administer gas was from a gasometer, dispensing with the rubber receiver.

M. M. Johnson, B. A., of New York, said he was largely interested in manufacturing apparatus for making and administering the gas, and would gladly receive any suggestion as to the best kind of apparatus. He was of opinion that gas should be passed through chemicals, to neutralize any poisonous acid that might be present.

Dr. Scott replied that the old way of passing gas through three wash bottles containing chemicals had not proved satisfactory; that chemicals would soon become charged with impurities generated by increased heat required in using three bottles. One bottle filled with water was all that was necessary, whatever else might be found most plausible was of little consequence, when actual practice showed that one bottle gave better gas than three.

Dr. J. B. Meacham, Brantford, said that he had succeeded very well with gas. He had formerly made it in the old way; but intended to use a gasometer, as he was satisfied it was the better way.

Dr. G. V. N. Relyea moved, seconded by Dr. Wood, that in the opinion of this Association, Dentists should not administer chloroform except in presence of a physician. Carried.

Moved by J. O'Donnell, seconded by W. C. Adams, that this Association considers the displaying of cases of mechanical Dentistry at doors as a means of attracting the public, and also of doggerel rhymes and high-sounding puffs of capability, as a species of quackery beneath the dignity of any respectable Dentist. The Recording Secretary stated that the Medical Council, which was in session in Ottawa, recently had passed a resolution approving of the passing of an Act to incorporate the Dental profession.

Drs. Berryman and Patullo, members of the Medical Council, were elected honorary members of the Association.

Moved by H. T. Wood, seconded by L. Lemon, that G. V. N. Relyea, C. S. Chittenden, F. G. Callender, and J. B. Meacham, be elected delegates to the American Dental Association. Carried.

